# SARS-CoV-2 Infection Causes Dopaminergic Neuron Senescence

**DOI:** 10.21203/rs.3.rs-513461/v1

**Published:** 2021-05-21

**Authors:** Yuling Han, Liuliu Yang, Tae Wan Kim, Manoj S. Nair, Oliver Harschnitz, Pengfei Wang, Jiajun Zhu, So Yeon Koo, Xuming Tang, Lauretta A. Lacko, Vasuretha Chandar, Yaron Bram, Tuo Zhang, Wei Zhang, Feng He, James Caicedo, Yaoxing Huang, Todd Evans, Paul van der Valk, Maarten J. Titulaer, Jochem K. H. Spoor, Robert L. Furler, Peter Canoll, James E. Goldman, Serge Przedborski, Robert E. Schwartz, David D. Ho, Lorenz Studer, Shuibing Chen

**Affiliations:** 1 Department of Surgery, Weill Cornell Medicine, 1300 York Ave, New York, NY, 10065, USA.; 2 The Center for Stem Cell Biology, Sloan-Kettering Institute for Cancer Research, New York, NY 10065, USA.; 3 Developmental Biology Program, Sloan-Kettering Institute for Cancer Research, New York, NY 10065, USA.; 4 Aaron Diamond AIDS Research Center, Columbia University Vagelos College of Physicians and Surgeons, New York, NY, 10032, USA.; 5 Neuroscience Graduate Program of Weill Cornell Graduate School of Biomedical Sciences, New York, NY, USA; 6 Division of Infectious Diseases, Department of Medicine, Weill Cornell Medicine, 1300 York Ave, New York, NY, 10065, USA.; 7 Division of Gastroenterology and Hepatology, Department of Medicine, Weill Cornell Medicine, 1300 York Ave, New York, NY, 10065, USA.; 8 Department of Physiology, Biophysics and Systems Biology, Weill Cornell Medicine, 1300 York Ave, New York, NY, 10065, USA. New York 10021, USA; 9 Genomic Resource Core Facility, Weill Cornell Medicine, New York, NY 10065, USA.; 10 Department of Neurology, Columbia University Irving Medical Center, Vagelos College of Physicians and Surgeons, Columbia University, New York, NY, 10032, USA; 11 Department of Pathology, Amsterdam University Medical Center, VU University Amsterdam, Amsterdam, The Netherlands; 12 Department of Neurology, Erasmus University Medical Center, Rotterdam, The Netherlands; 13 Department of Neurosurgery, Erasmus University Medical Center, Rotterdam, The Netherlands; 14 Department of Pathology and Cell Biology, Columbia University Irving Medical Center, Vagelos College of Physicians and Surgeons, Columbia University, New York, NY, 10032, USA; 15 Department of Neuroscience, Columbia University, New York, NY, 10032, USA

**Keywords:** SARS-CoV-2, midbrain dopamine, neuron senescence

## Abstract

COVID-19 patients commonly present with neurological signs of central nervous system (CNS)^1–3^ and/or peripheral nervous system dysfunction^4^. However, which neural cells are permissive to infection by severe acute respiratory syndrome coronavirus 2 (SARS-CoV-2) has been controversial. Here, we show that midbrain dopamine (DA) neurons derived from human pluripotent stem cells (hPSCs) are selectively permissive to SARS-CoV-2 infection both *in vitro* and upon transplantation *in vivo*, and that SARS-CoV-2 infection triggers a DA neuron inflammatory and cellular senescence response. A high-throughput screen in hPSC-derived DA neurons identified several FDA approved drugs, including riluzole, metformin, and imatinib, that can rescue the cellular senescence phenotype and prevent SARS-CoV-2 infection. RNA-seq analysis of human ventral midbrain tissue from COVID-19 patients, using formalin-fixed paraffin-embedded autopsy samples, confirmed the induction of an inflammatory and cellular senescence signature and identified low levels of SARS-CoV-2 transcripts. Our findings demonstrate that hPSC-derived DA neurons can serve as a disease model to study neuronal susceptibility to SARS-CoV-2 and to identify candidate neuroprotective drugs for COVID-19 patients. The susceptibility of hPSC-derived DA neurons to SARS-CoV-2 and the observed inflammatory and senescence transcriptional responses suggest the need for careful, long-term monitoring of neurological problems in COVID-19 patients.

Abnormal neurological manifestations are increasingly recognized in patients with COVID-19 which most commonly include anosmia, dysgeusia, and headache followed by seizure, stroke, and acute inflammatory polyradiculoneuropathy also known as Guillain-Barre syndrome^5^. Furthermore, an increased risk for additional neurological and psychiatric disorders has been reported in a large retrospective cohort at 6 months post diagnosis^6^. Up-to-now, however, little is known about which neural cell types or neuronal subtypes are permissive to infection by the severe acute respiratory syndrome coronavirus 2 (SARS-CoV-2). Recently, we developed a human pluripotent stem cell (hPSC)-derived organoid/cell-based platform to evaluate the tropism of SARS-CoV-2. Using this platform, we found that hPSC-derived midbrain dopamine (DA) neurons, representing one of the main target of the neurodegenerative process in Parkinson’s disease (PD), are permissive to SARS-CoV-2 infection^7^. Conversely, under identical experimental conditions, we found that hPSC-derived cortical neurons are not permissive to SARS-CoV-2 infection^8^, supporting the notion that not all neuronal populations are equally permissive to viral infection. Here, we set out to define how DA neurons respond to SARS-CoV-2 infection and to determine the molecular changes induced by SARS-CoV-2 infection.

To examine the impact of SARS-CoV-2 infection on DA neurons, Nurr1:GFP reporter hPSCs were differentiated toward a DA neuron fate using a previously established strategy^8, 9^. The resulting DA neurons were validated by the expression of Nurr1:GFP, TH, and FOXA2 (Extended Data Fig. 1a). Expression of ACE2, the SARS-CoV-2 receptor, in Nurr1:GFP^+^ DA neurons was validated by immunostaining (Extended Data Fig. 1b). The permissiveness to SARS-CoV-2 entry was further confirmed using a vesicular stomatitis ΔG-luciferase virus pseudotyped with the SARS-CoV-2 Spike protein incorporated at the surface of the viral particle (SARS-CoV-2-entry virus)^10, 11^. Robust luciferase activity was readily detected in DA neurons infected with this SARS-CoV-2-entry virus (Extended Data Fig. 1c). Immunostaining further validated the expression of luciferase in DA neurons (Extended Data Fig. 1d).

To generate an *in vivo* model to study SARS-CoV-2 infection in human DA neurons, hPSC-derived DA neurons were transplanted under the anterior chamber of the eye, which allows the convenient observation of the grafted cells (Fig. 1a). One week after transplantation, immunohistochemistry was performed to validate ACE2 expression on Nurr1:GFP^+^ DA neurons (Fig. 1b). At 24 hours after intraocular inoculation with SARS-CoV-2-entry virus (1×10^4^ PFU), luciferase was mainly detected in Nurr1: GFP^+^ DA neurons as indicated by immunofluorescence staining (Fig. 1c). This suggests that hPSC-derived DA neurons are permissive to SARS-CoV-2 infection when exposed to the virus *in vivo*. Due to the limitations of BSL-3 animal protocols, this transplantation model was not further applied to test an intranasal infection route.

Next, hPSC-derived DA neurons were infected *in vitro* with SARS-CoV-2 (USA-WA1/2020, MOI=0.2). At 48 hours post infection (hpi), qRT-PCR analysis using primers targeting subgenomic N transcripts confirmed that significant amounts of viral replication could be detected at the RNA level in infected hPSC-derived DA neurons (Fig. 1d). Immunostaining for the SARS-N protein confirmed robust SARS-CoV-2 infection of DA neurons (Fig. 1e). Finally, transmission electron microscopy was used to detect the presence of viral particles in SARS-CoV-2 infected hPSC-derived DA neurons (Fig. 1f). Overall, these *in vitro* and *in vivo* experiments confirm that human hPSC-derived DA neurons are permissive to SARS-CoV-2 and support productive infection.

RNA-seq analysis was applied to compare mock-infected or SARS-CoV-2 infected hPSC-derived DA neurons. Robust viral infection was detected in SARS-CoV-2 infected DA neurons (Fig. 1g). Moreover, plotting these datasets by principal component analysis (PCA, Fig. 1h) and clustering analysis (Fig. 1i) demonstrated that the infected DA neurons occupied a distinct transcriptional space compared to mock-infected DA neurons. In contrast, no obvious transcriptional changes were observed following SARS-CoV-2 exposure of hPSC-derived cortical neurons. In hPSC-derived DA neurons, we next analyzed DA neuron marker expression and found that levels of *FOXA2* and *NURR1* were decreased in SARS-CoV-2 infected samples (Fig. 1j). In particular, markers of the A9 DA neurons ‒ the subtype of ventral midbrain DA neurons most affected in PD ‒ such as *LMO3, DKK3*, and *ALDH1A1*^12^, were significantly downregulated in SARS-CoV-2 infected cells (Fig. 1j). Quantitative RNA in situ hybridization further confirmed the decrease of A9 markers including *LMO3* expression following SARS-CoV-2 infection, indicating an increased vulnerability of human DA neurons expressing A9 subtype specific markers to SARS-CoV-2 infection (Fig. 1k, 1l).

Volcano plots and heatmap of SARS-CoV-2 infected versus mock-infected hPSC-derived DA neurons showed robust induction of chemokine and cytokine transcripts, including *BMP2, CCL2, CCL20, CCL25, CXCL1, CXCL14, IGFBP7, IL11, IL12A, IL1A, IL1B, IL34, IL5*, and *TNFRSF1A* (Fig. 2a, 2b). Those transcriptional changes were specific to DA neuron cultures and again not observed in cortical neuron cultures, in line with our previous work showing a lack of susceptibility of cortical neurons to SARS-CoV-2^7^. In infected DA neurons, inflammation-associated genes were also upregulated in SARS-CoV-2 infected DA neurons (Fig. 2c). Ingenuity Pathway Analysis highlighted the senescence pathway as the top regulated pathway in SARS-CoV-2 infected DA neurons (Fig. 2d), a finding further corroborated by the upregulated expression of key genes involved in the senescence pathway (Fig. 2e). Beta-galactosidase (Beta-Gal), a biomarker of cellular senescence^8^, was also upregulated in SARS-CoV-2 infected hPSC-derived DA neurons (Fig. 2f, 2g). qRT-PCR analysis was performed for examining the expression of senescence-pathway associated genes, including *IGFBP7* and *LAMIN B1*. Consistent with senescence-associated regulation of those two genes in previous studies^8, 13^, *IGFBP7* was significantly upregulated in SARS-CoV-2 infected DA neurons while *LAMIN B1* was significantly downregulated (Fig. 2h). Finally, transmission electron microscopy detected lipofuscin in SARS-CoV-2 infected DA neurons as an additional senescence-associated marker of DA neurons^15^ (Fig. 2i). The induction of DA neuron senescence and evidence of increased vulnerability of human A9 DA neurons suggest that SARS-CoV-2 infection could serve as a potential degenerative trigger for DA neurons.

To identify drug candidates that may protect from SARS-CoV-2-induced senescence, we screened DA neurons against a library of FDA-approved drugs supplied at 10 μM. Six hours post-treatment, DA neurons were infected with SARS-CoV-2 at MOI=0.2. At 72 hpi, hPSC-derived DA neurons were analyzed for levels of Beta-Gal. Compounds with a Z score <−2 were defined as primary hit drugs (Fig. 3a). The hits were further evaluated for potency and cytotoxicity at different concentrations. Three drugs, riluzole (Fig. 3b, 3e), metformin (Fig. 3c, 3f), and imatinib (EC_50_=3.25 μM, CC_50_=17.14 μM, Fig. 3d, 3g), reduced Beta-Gal activity in a dose-dependent manner without inducing cytotoxicity. Furthermore, wells treated with either 10 μM riluzole, 50 μM metformin, or 10 μM imatinib showed a significant decrease in the total number of Beta-Gal^+^ cells as compared to DMSO treatment (Fig. 3h, 3i). Finally, qRT-PCR analysis showed a decrease of the senescence-pathway associated gene *IGFBP7* and an upregulation of *LAMIN B1* for each of the three drugs (Fig. 3j).

The lead compounds might decrease senescence by blocking SARS-CoV-2 infection or rescuing SARS-CoV-2-induced senescence. To distinguish between these possibilities, DA neurons were again treated with 10 μM riluzole, 50 μM metformin, or 10 μM imatinib and infected with SARS-CoV-2. At 48 hpi, qRT-PCR analysis demonstrated that riluzole, metformin, and imatinib all decreased viral RNA (Fig. 3k), a finding further validated by immunostaining using an antibody against the SARS-CoV-2 Nucleocapsid protein (Fig. 3l, 3m). Interestingly, we identified imatinib previously as an anti-SARS-CoV-2 drug in hPSC-lung organoids^14^.

RNA-seq analysis was applied to determine the transcriptional changes induced by the drug candidates versus DMSO in DA neurons upon SARS-CoV-2 infection. Plotting these datasets by PCA (Fig. 3n) and by performing clustering analysis (Fig. 3o) demonstrated that DA neurons treated with drug candidates occupied a distinct transcriptional space compared to DMSO-treated control DA neurons. Importantly, the genes involved in senescence pathway were downregulated in riluzole, metformin or imatinib treated DA neurons (Fig. 3p).

A key question is whether the selective vulnerability of hPSC-derived DA neurons and the resulting senescence and inflammatory responses are reflected in any cognate changes in the brain of human COVID-19 patients. To directly probe the human substantia nigra, we performed RNA-seq analysis on RNA isolated from formalin-fixed paraffin-embedded (FFPE) autopsy samples from three COVID-19 patients and three age-matched controls. Remarkably, the same transcriptional signatures identified in SARS-CoV-2 infected DA neurons *in vitro* (Fig. 2b–d), were observed in COVID-19 autopsy samples, including the induction of chemokine/cytokine (Fig. 4a), inflammation (Fig. 4b), and senescence-associated (Fig. 4c) genes. These data provide evidence for an ongoing inflammatory and senescence response within the substantia nigra of COVID-19 patients despite the lack of overt neuropathological changes^15^. The RNA-seq data also showed expression of several SARS-CoV-2 transcripts across 6 ventral midbrain samples from COVID-19 patients, compatible with the presence of virus (Fig. 4d). However, we also detected very low levels of viral RNA by qRT-PCR in frozen tissue samples from other brain regions from these same autopsies which could potentially represent virus in leptomeningeal or intracerebral vessels^15^. Our findings on the selective vulnerability of hPSC-derived DA neurons *in vitro*, and the associated inflammatory and cell senescence responses observed in DA neurons *in vitro* and COVD-19 patient samples *in vivo* argue that these results may be of clinical relevance.

Advancements in hPSC-technology allow for the study of host-virus interactions in human, disease-relevant cells^16^. Recent studies using hPSC-derived organoid models have established that choroid plexus cells within the CNS are highly susceptible to SARS-CoV-2 infection^17, 18^. However, the tropism of SARS-CoV-2 for neurons has remained controversial^17–19^. Here, we report that SARS-CoV-2 can infect hPSC-derived DA neurons and triggers cellular senescence. Our previous work indicates that senescence of DA neurons can function as a contributing factor in PD pathogenesis^8^. As DA neuron dysfunction is also linked to lethargy and anhedonism^20^, its role in the post-COVID lethargy/syndrome may deserve further study. The FDA-approved drugs riluzole, metformin, and imatinib, shown here to block SARS-CoV2-mediated DA neuron senescence, could potentially be repurposed as COVID-19 therapeutics. While imatinib was also identified to block SARS-CoV-2 entry in our hPSC-derived lung organoid-based screen^14^, riluzole has not been previously linked to SARS-CoV-2 infection. The use of metformin has been associated with a decrease in the mortality of COVID-19 patients with obesity and/or type 2 diabetes^21, 22^. Overall, our data highlight DA neurons as a possible target for SARS-CoV-2 infection, which in turn may trigger an inflammatory and cellular senescence response in the substantia nigra. While we observed a comparable inflammatory and senescence signature in SARS-CoV2 infected hPSC-derived DA neuron cultures *in vitro* and in autopsy samples *in vivo* we cannot exclude the possibility that other cell types such as astrocytes or microglia or other pathological changes such as hypoxic state could contribute to the inflammatory and senescence signatures detected in the substantia nigra samples. Furthermore, microglial activation in the brainstem seems to be more severe than in other regions which could contribute to a possible dysfunction of DA neuron. Given our findings, we posit that over the coming years there is a need to closely monitor COVID-19 patients for an increased risk of developing PD-related symptoms.

## METHOD

### Construction of Nurr1:GFP hESCs.

Generation of Nurr1::GFP hESC line was previously described^8^. Briefly, stop codon of endogenous NR4A2 (Nurr1) was replaced by EGFP expression cassette (P2A-H2B-PgkPuro) by using a CRISPR/CAS9-mediated knock-in approach. The resulting *NURR1:GFP*^+^ cells almost express TH (a mature mDA marker; 98%) based on single cell qRT-PCR^8^.

### hESC differentiation toward DA neurons.

Midbrain dopaminergic neuron differentiation were performed using H9 hESCs, which include Nurr1: GFP hESC. hESCs were grown on VTN-N (Thermo Fisher Scientific)-coated 6-well plates in E8-essential medium. Cells were maintained at 37°C, 5% CO_2_. hESCs were differentiated with an optimized protocol from a previously reported study^8,23^.

### SARS-CoV-2-entry Viruses.

Recombinant Indiana VSV (rVSV) expressing SARS-CoV-2 spikes were generated as previously described^19^. HEK293T cells were grown to 80% confluency before transfection with pCMV3-SARS-CoV-2-spike (kindly provided by Dr. Peihui Wang, Shandong University, China) using FuGENE 6 (Promega). Cells were cultured overnight at 37°C with 5% CO2. The next day, medium was removed and VSV-G pseudo-typed ΔG-luciferase (G*ΔG-luciferase, Kerafast) was used to infect the cells in DMEM at a MOI of 3 for 1 hour before washing the cells with 1×DPBS three times. DMEM supplemented with anti-VSV-G antibody (I1, mouse hybridoma supernatant from CRL-2700; ATCC) was added to the infected cells and they were cultured overnight as described previously^24^. The next day, the supernatant was harvested and clarified by centrifugation at 300 g for 10 minutes and aliquots stored at −80°C.

hPSC-derived DA neurons were seeded in 24-well plates, SARS-CoV-2-entry virus was added at the indicated MOIs for 1 hour. Then, the cells were cultured at 37°C with 5% CO2. At 24 hpi, cells were fixed for immunohistochemistry or harvested for luciferase assay following the Luciferase Assay System protocol (E1501, Promega)

### SARS-CoV-2 Virus infections.

SARS-CoV-2, isolate USA-WA1/2020 was obtained from World Reference Center for Emerging Viruses and Arboviruses located at University of Texas, Medical Branch via the CDC. SARS-CoV-2 was propagated in Vero E6 cells (ATCC) in EMEM supplemented with 10% FCS, 1 mM Sodium Pyruvate and 10 mM HEPES as described previously^24^.

SARS-CoV-2 infections of hPSC-derived DA neurons were performed in the culture media at the indicated MOIs at 37°C. At the indicated hpi, cells were washed three times with PBS. For RNA analysis cells were lysed in TRIzol (Invitrogen). For immunofluorescence staining cells were fixed in 4% formaldehyde for 60 min at room temperature.

All work involving live SARS-CoV-2 was performed in the CDC/USDA-approved BSL-3 facility at Aaron Diamond AIDS Research Center located at Columbia University.

### Anterior eye chamber transplantation.

hESCs-derived DA neurons were resuspended in 10 μL medium and injected into the anterior eye chamber of 6 to 8-week-old male NSG mice. 1-week post-transplantation, SARS-CoV-2-entry virus was inoculated locally at 1×10^4^ PFU. At 24 hpi, the mice were euthanized and used for immunohistochemistry analysis.

All animal work was performed under the approval of Institutional Animal Care and Use Committee (IACUC) at Weill Cornell Medicine.

### Immunohistochemistry.

Histology on tissues from mice was performed on frozen sections from xenografts. Tissues were fixed in 4% paraformaldehyde and transferred to 30% sucrose, followed by snap freezing in O.C.T (Fisher Scientific, Pittsburgh, PA). Living cells in culture were directly fixed in 4% paraformaldehyde for 25 min, followed with 15 min permeabilization in 0.1% Triton X-100. For immunofluorescence, cells or tissue sections were immuno-stained with primary antibodies at 4°C overnight and secondary antibodies at RT for 1h. The information for primary antibodies and secondary antibodies is provided in Extended Data Table 2. Nuclei were counterstained by DAPI.

### X-Galactosidase Staining.

The identification of senescent cells is based on an increased level of β-galactosidase activity. The assay followed Senescence β-Galactosidase Staining Kit (#9860, CST).

### qRT-PCR.

Total RNA samples were prepared from cells and DNase I treated using TRIzol according to the manufacturer’s instructions. To quantify viral replication, measured by the expression of sgRNA transcription of the viral N gene, one-step quantitative real-time PCR was performed using SuperScript III Platinum SYBR Green One-Step qRT-PCR Kit (Invitrogen) with primers specific for the TRS-L and TRS-B sites for the N gene as well as ACTB as an internal reference. Quantitative real-time PCR reactions were performed on an Applied Biosystems QuantStudio 6 Flex Real-Time PCR Instrument (ABI). Delta-delta-cycle threshold (ΔΔCT) was determined relative to ACTB levels and normalized to mock infected samples. Error bars indicate the standard deviation of the mean from three biological replicates. The sequences of primers/probes are provided in Extended Data Table 3.

### RNA-Seq before and following viral infections.

Cell infections were performed at the described MOI in DMEM supplemented with 0.3% BSA, 4.5 g/L D-glucose, 4 mM L-glutamine and 1 μg/ml TPCKtrypsin and harvested 24 hpi. Total RNA was extracted in TRIzol (Invitrogen) according to the manufacturer’s instructions. RNAseq libraries of polyadenylated RNA were prepared using the TruSeq Stranded mRNA Library Prep Kit (Illumina) according to the manufacturer’s instructions and sequenced on an Illumina NextSeq 500 platform. The resulting single end reads were checked for quality (FastQC v0.11.5) and processed using the Digital Expression Explorer 2 (DEE2)^25^ workflow. Adapter trimming was performed with Skewer (v0.2.2)^26^. Further quality control done with Minion, part of the Kraken package^27^. The resultant filtered reads were mapped to human reference genome GRCh38 using STAR aligner^28^ and gene-wise expression counts generated using the “-quantMode GeneCounts” parameter. BigWig files were generated using the bamCoverage function in deepTools2 (v.3.3.0)^29^.

For RNA prep with human exome enrichment, total RNA samples were prepared from formalin-fixed and paraffin-embedded autopsy ventral midbrain tissues followed by DNaseI treatment using manufacturer’s instructions (Qiagen RNeasy FFPE kit Cat# 73604). 100 ng total RNA was prepared using NEB Next Ultra II RNA Library Prep Kit without polyA selection or RNA depletion, then the libraries were enriched with twist human exome probes and reagents.

For RNA prep with Covid 19 panel enrichment, 100ng total RNA was prepared using NEB Next Ultra II RNA Library Prep Kit without polyA selection or RNA depletion, then the libraries were enriched with IDT covid 19 Capture Panel probes and reagents.

For analysis, the salmon index was built using the human transcriptome GRCh38.p13. The index is a structure that salmon uses to quasi-map RNA-seq reads during quantification. Then, the fastq format RNA-seq raw data was quantified with salmon. The quantification results were analyzed using the tximport package to import salmon’s transcript-level quantifications and were aggregated to the gene level for gene-level differential expression analysis using the DESeq2 package.

### In situ hybridization.

Adherent cells plated in a glass-bottom plate are fixed and permeabilized and stained for a protein of interest (TH; tyrosine hydroxylase) in order to locate RNA puncta signals within a mature DA neuron. Following protein detection, a fluorescent *in situ* hybridization (FISH) and branched DNA amplification technology is used to amplify the signal detection of an RNA transcript. In the first step, a gene-specific oligonucleotide target probe binds to the target RNA sequence. Signal amplification is then achieved through a series of sequential hybridization steps. After two sequential amplifying steps, a fluorescent dye is introduced to hybridize to their corresponding amplifier molecules. RNA signals in dots are visualized using confocal microscopy with 63X oil lenses. All the images in z-stacks were projected and obtained using Imaris software. Projected images were analyzed for quantification.

### High Throughput Chemical Screening.

hPSC-derived DA neurons were cultured in 384-well plates at 10,000 cells/50 μl medium/well until Day 40. Compounds from an in-house FDA-approved drug library (Prestwick) were added at 10 μM. DMSO treatment was used as a negative control. hPSC-derived DA neurons were further infected with SARS-CoV-2 (MOI=0.1). After 72 hpi, hPSC-derived DA neurons were harvested for β-galactosidase assay using Senescence β-Galactosidase Staining Kit (#9860, CST) protocol.

To calculate EC50 and CC50, cells were stained with β-Galactosidase Staining Kit and normalized to DMSO-treated condition. To calculate CC50, the cell survival was monitored by DAPI and normalized to DMSO-treated condition. The efficacy and cytotoxicity curves were calculated using Prism GraphPad Prism 7.0.

### Human Studies.

The brain samples were from the midbrain and the frontal cortex. They came from a prospective autopsy cohort study, conducted at the Columbia University Presbyterian Hospital and approved by its institutional review board. Informed consent for complete autopsy (including the brain, for which separate and explicit consent was asked) was obtained. In addition, additional samples came from a prospective autopsy cohort study, conducted at Amsterdam University Medical Center, the Netherlands (two locations) and approved by its institutional review board. For both sites, informed consent for complete autopsy (including the brain, for which separate and explicit consent was asked) was obtained. The brain samples were fixed in 4% formaldehyde and routinely processed for paraffin-embedding. Experiments using samples from human subjects were conducted in accordance with local regulations and with the approval of the institutional review board at the Weill Cornell Medicine under protocol METC 2020.167.

## Quantification and Statistical analysis.

N=3 independent biological replicates were used for all experiments unless otherwise indicated. n.s. indicates a non-significant difference. *P*-values were calculated by unpaired two-tailed Student’s t-test unless otherwise indicated. *p<0.05, **p<0.01 and ***p<0.001.

## Extended Data

**Extended Data Figure 1.**
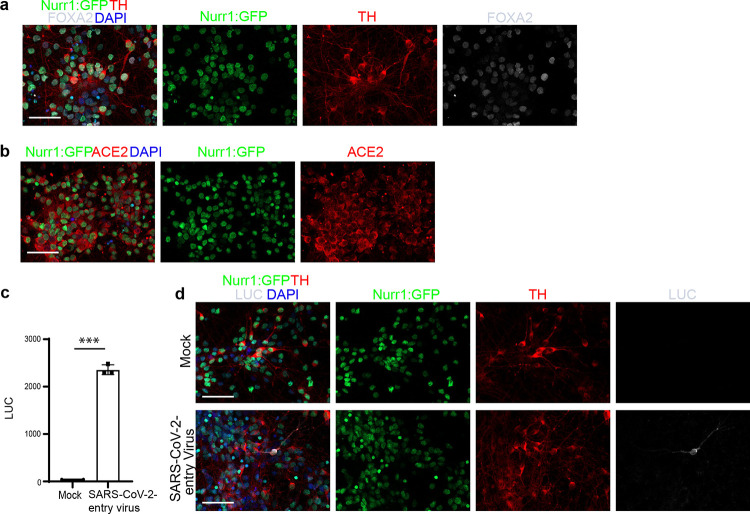
hPSC-derived dopaminergic cells can be infected by SARS-CoV-2 pseudo-entry virus. **a,** Representative confocal images of hPSC-derived DA neurons stained with antibodies recognizing Nurr1-GFP, TH or FOXA2. Scale bar=50μm. **b,** Representative confocal images of hPSC-derived DA neurons stained with ACE2 antibody. Scale bar=50μm. **c,** Luciferase activity in lysates from hPSC-derived DA neurons at 24 hpi following exposure to SARS-CoV-2-entry virus at MOI=0.01. **d,** Representative confocal images of hPSC-derived DA neurons infected with SARS-CoV-2-entry virus (MOI=0.01) at 24 hpi using antibodies against luciferase and DA markers. Scale bar=50μm. Data was presented as mean ± STDEV. *P* values were calculated by unpaired two-tailed Student’s t test. ***P < 0.001.

**Extended Data Table 1.** Patient information.

Patient ID	Gender	Age
COVID_1	Male	66
COVID_4	Female	78
COVID_5	Female	80
COVID_6	Male	80
COVID_8	Male	64
COVID_9	Male	59
Healthy_1	Male	36
Healthy_2	Male	60
Healthy_3	Female	61

**Extended Data Table 2.** Antibodies used for immunocytochemistry, intracellular flow cytometry analysis and western blotting analysis.

Usage	Antibody	Clone #	Host	Catalog *#*	Vendor	Dilution
Immunocytoche mistry	ACE2	Polyclonal	Rabbit	ab15348	Abcam	1:500
Immunocytoche mistry	Firefly luciferase Monoclonal Antibody (CS 17)	CS 17	Mouse	35–6700	Thermo Fisher Scientifi c	1:200
Immunocytoche mistry	Goat polyclonal anti-FOXA2	Polyclonal	Goat	AF2400	R&D Systems	1:250
Immunocytoche mistry	Anti-Tyrosine Hydroxylase antibody - Neuronal Marker	Polyclonal	Rabbit	ab112	Abcam	1:500
Immunocytoche mistry	Human/Mouse Tyrosine Hydroxylase Antibody	779427	Mouse	MAB7566	R&D Systems	1:200
Immunocytoche mistry	Anti-FOXA2 Antibody	M-20	Goat	sc-6554	Santa Cruz	1:150
Immunocytoche mistry	Donkey anti-Mouse IgG (H+L) Cross-Adsorbed Secondary Antibody, Alexa Fluor 488	Polyclonal	Donkey	#A-21202	Thermo Fisher Scientifi c	1:500
Immunocytoche mistry	Donkey anti-Rabbit IgG (H+L) Secondary Antibody, Alexa Fluor 594	Polyclonal	Donkey	#A-21207	Thermo Fisher Scientifi c	1:500
Immunocytoche mistry	Donkey anti-Goat IgG (H+L) Cross-Adsorbed Secondary Antibody, Alexa Fluor 647	Polyclonal	Donkey	#A-21447	Thermo Fisher Scientifi c	1:500
Immunocytoche mistry	Donkey anti-Goat IgG Secondary Antibody, Alexa Fluor 594	Polyclonal	Donkey	A32816	Thermo Fisher	1:500
Immunocytoche mistry	Donkey anti-Rabbit IgG Secondary Antibody, Alexa Fluor 647	Polyclonal	Donkey	A32795	Thermo Fisher	1:500

**Extended Data Table 3.** Primers used for qRT-PCR.

Primer name	Sequence
*ACTB-Forward*	*CGTCACCAACTGGGACGACA*
*ACTB-Reverse*	*CTTCTCGCGGTTGGCCTTGG*
*SARS-CoV-2-TRS-L*	*CTCTTGTAGATCTGTTCTCTAAACGAAC*
*SARS-CoV-2-TRS-N*	*GGTCCACCAAACGTAATGCG*
*LaminB1-F*	*AAGCATGAAACGCGCTTGG*
*LaminB1-R*	*AGTTTGGCATGGTAAGTCTGC*
*IGFBP7-F*	*ATCCCGACACCTGTCCTCAT*
*IGFBP7-R*	*CCCAGCCAGTTACTTCATGCT*

## Figures and Tables

**Figure 1.**
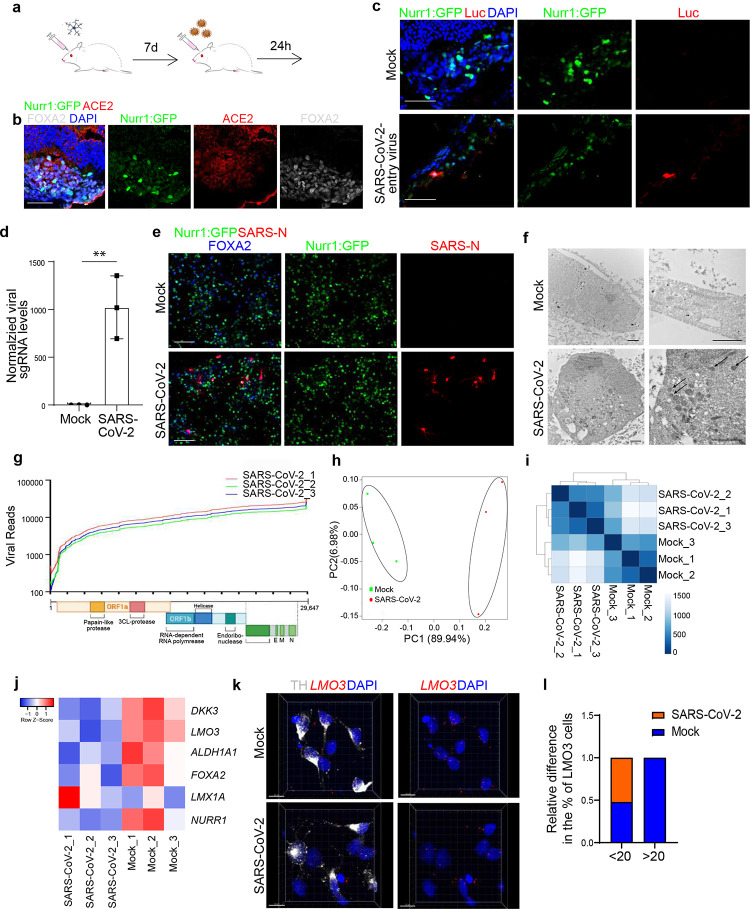
hPSC-derived DA neurons, and in particular A9 DA neurons, are permissive to SARS-CoV-2 infection. **a,** Schematic for *in vivo* infection. **b,** Representative confocal image of DA neuron xenograft stained with antibodies against ACE2 and Nurr1-GFP. Scale bar=50μm. **c,** Representative confocal image of DA neuron xenograft at 24 hpi stained with antibodies against Luc and Nurr1-GFP. Scale bar=60μm. **d**. qRT-PCR analysis of total RNA extracted from hPSC-derived DA neurons at 48 hpi of SARS-CoV-2 infection (MOI=0.2) for viral N sgRNA. The graph depicts the mean sgRNA level normalized to *ACTB*. **e,** Representative confocal images of hPSC-DA neurons infected with SARS-CoV-2 (MOI=0.1) at 72 hpi using antibodies against SARS-CoV-2 Nucleocapsid protein (SARS-N) and markers for DA neurons. Scale bar=50μm. **f,** Transmission electron microscope (TEM) images of DA neurons at 72 hpi of SARS-CoV-2 (MOI=1.0). Arrows point to SARS-CoV-2 viral particles. Right panel: Zoom in images. Scale bar=1μm. **g,** RNA-seq read coverage of the viral genome in infected hPSC-derived DA neurons at 48 hpi (MOI=0.2). The schematic below depicts the SARS-CoV-2 genome and was created using BioRender. **h,** PCA plot of gene expression profiles from mock infected and SARS-CoV-2 infected hPSC-derived DA neurons at 48 hpi (MOI=0.2). **i,** Clustering analysis of mock or SARS-CoV-2 infected hPSC-derived DA neurons at 48 hpi (MOI=0.2). **j,** Heatmap of DA neurons and A9 DA neuron marker genes expression levels in mock or SARS-CoV-2 infected hPSC-derived DA neurons at 48 hpi (MOI=0.2). **k, l,** Fluorescence in situ hybridization (k) and quantification (l) of A9 DA marker, *LMO3*, in mock or SARS-CoV-2 infected hPSC-derived DA neurons at 48 hpi (MOI=0.2) Scale bar=10μm. N=3 independent biological replicates. Data was presented as mean ± STDEV. *P* values were calculated by unpaired two-tailed Student’s t test. **P < 0.01.

**Figure 2.**
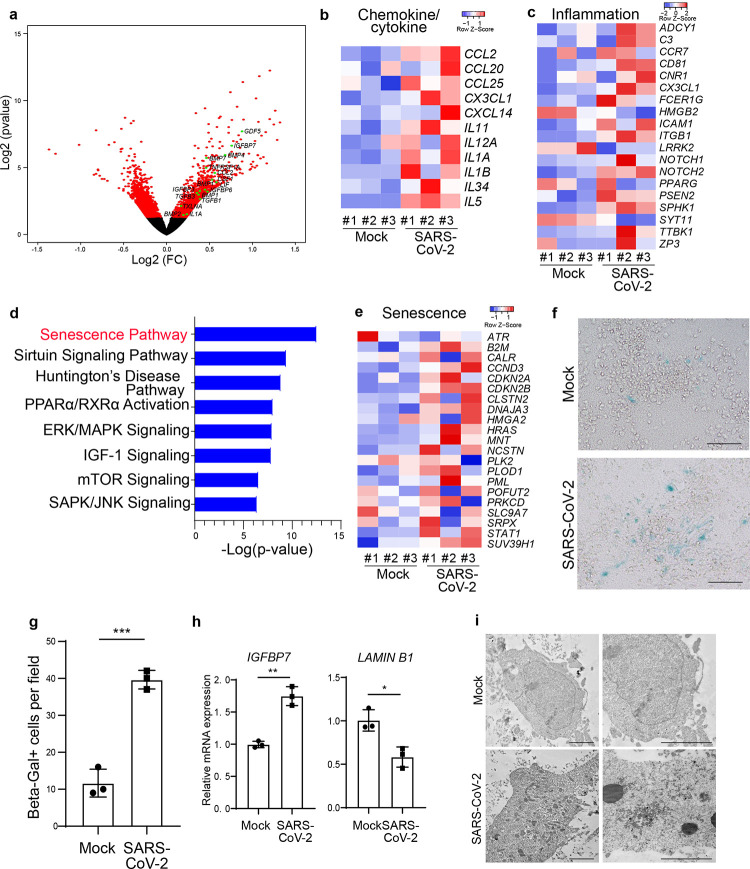
SARS-CoV-2 infection induces senescence of DA neurons. **a,** Volcano plot indicating differentially expressed genes in mock or SARS-CoV-2 infected hPSC-derived DA neurons at 48 hpi (MOI=0.2). Differentially expressed genes (p-adjusted value < 0.05) are indicated in red. Non-significant differentially expressed genes with a log2 (Fold Change) > 0.5 are indicated in black. **b, c,** Heatmap of chemokine/cytokines (b) and inflammation associated genes (c) in mock or SARS-CoV-2 infected hPSC-derived DA neurons at 48 hpi (MOI=0.2). **d,** IPA analysis of differentially expressed genes in a. **e,** Heatmap of senescence associated genes in mock or SARS-CoV-2 infected hPSC-derived DA neurons at 48 hpi (MOI=0.2). **f, g,** Beta-Gal staining (f) and quantification (g) of mock or SARS-CoV-2 infected hPSC-derived DA neurons at 72 hpi (MOI=0.1). Scale bar=75μm. **h.** qRT-PCR analysis of senescence related genes of mock or SARS-CoV-2 infected hPSC-derived DA neurons at 48 hpi (MOI=0.2). **i.** TEM images of mock or SARS-CoV-2 infected hPSC-derived DA neurons at 72 hpi (MOI=1.0). Scale bar=2μm. N=3 independent biological replicates. Data was presented as mean ± STDEV. *P* values were calculated by unpaired two-tailed Student’s t test. *P < 0.05, **P < 0.01, and ***P < 0.001.

**Figure 3.**
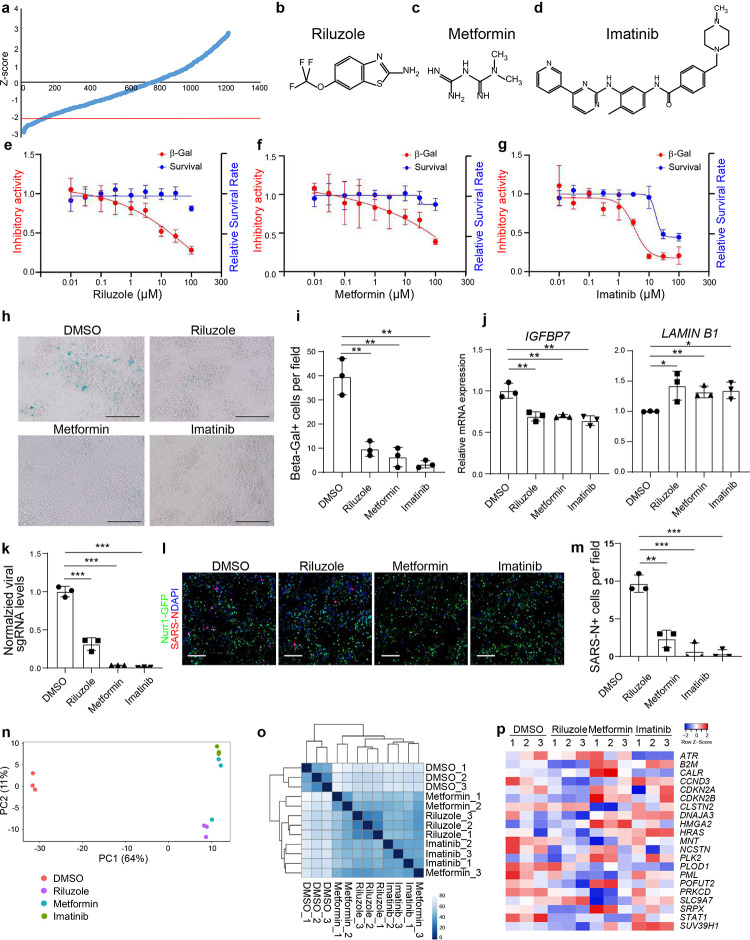
Riluzole, metformin, and imatinib rescue SARS-CoV-2 induced senescence of DA neurons. **a,** Primary screening results. X-axis is the compound number. Y axis is the Z-score. Red line is Z-score <−2, which means the luminescent signal is lower than average-2xSTDEV. **b-d,** Chemical structures of riluzole (b), metformin (c), and imatinib (d). **e-g,** Efficacy and cytotoxicity curves of riluzole (e), metformin (f), and imatinib (g). **h, i,** Beta-Gal staining (h) and the quantification (i) of DMSO or drug candidate-treated hPSC-derived DA neurons at 72 hpi upon SARS-CoV-2 infection (MOI=0.1). Scale bar=100μm. **j,** qRT-PCR analysis of senescence related genes of DMSO or drug candidate-treated hPSC-derived DA neurons at 48 hpi upon SARS-CoV-2 infection (MOI=0.1). **k,** qRT-PCR analysis of total RNA extracted from DMSO or drug candidate-treated hPSC-derived DA neurons at 48 hpi upon SARS-CoV-2 infection (MOI=0.1) for viral N sgRNA. The graph depicts the mean sgRNA level normalized to *ACTB*. **l, m,** Representative confocal images (l) and quantification (m) of DMSO or drug candidate-treated hPSC-derived DA neurons at 72 hpi upon SARS-CoV-2 infection (MOI=0.1) using antibodies against SARS-CoV-2 Nucleocapsid protein (SARS-N) and markers for DA neurons. Scale bar=100μm. **n, o,** PCA plot of gene expression profiles (n) and clustering analysis (o) of DMSO or drug candidate-treated hPSC-derived DA neurons at 48 hpi upon SARS-CoV-2 infection (MOI=0.1). **p,** Heatmap of senescence associated genes of DMSO or drug candidate-treated hPSC-derived DA neurons at 48 hpi upon SARS-CoV-2 infection (MOI=0.1). N=3 independent biological replicates. Data was presented as mean ± STDEV. *P* values were calculated by unpaired two-tailed Student’s t test. *P < 0.05, **P < 0.01, and ***P < 0.001.

**Figure 4.**
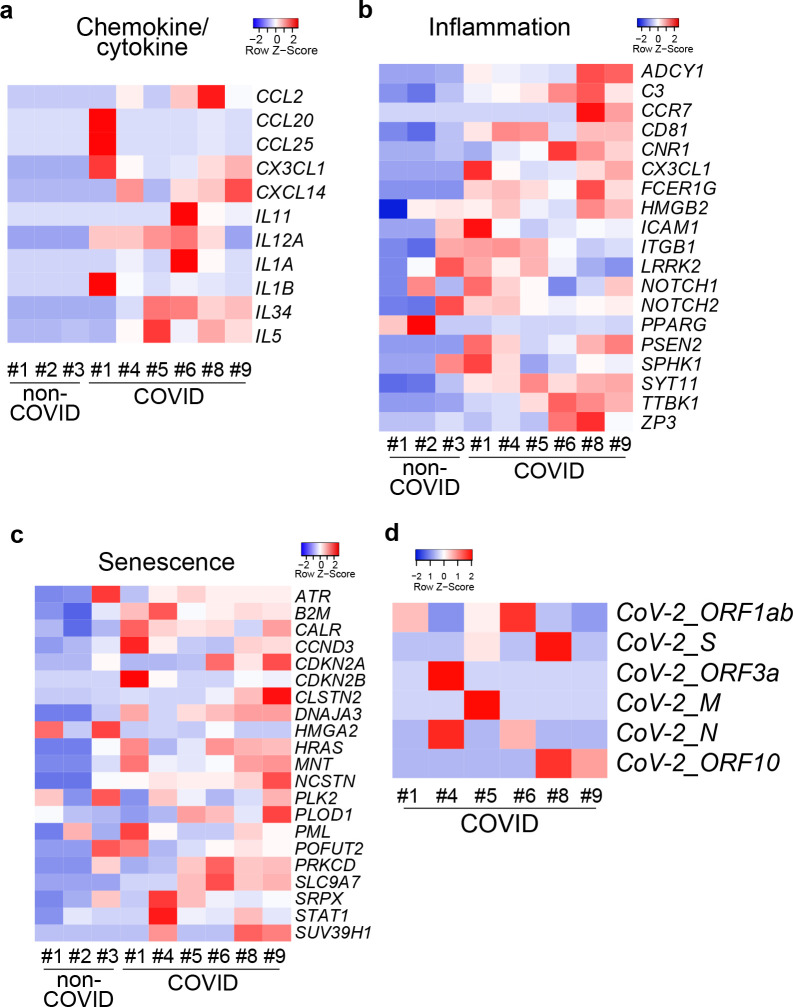
SARS-CoV-2 is detected in autopsy substantia nigra samples of COVID-19 patients. **a-c,** Heatmap of chemokine/cytokine (a), inflammation associated genes (b) and senescence associated genes (c) in the autopsy substantia nigra sections of COVID-19 patients versus non-COVID-19 patients. (N=6 COVID-19 patients; N=3 non-COVID-19 patients). **d,** Heatmap of viral transcripts in autopsy substantia nigra sections of COVID-19 patients.
